# Transition of amyloid/mutant p53 from tumor suppressor to an oncogene and therapeutic approaches to ameliorate metastasis and cancer stemness

**DOI:** 10.1186/s12935-022-02831-4

**Published:** 2022-12-26

**Authors:** Shinjinee Sengupta, Shaikh Maryam Ghufran, Aqsa Khan, Subhrajit Biswas, Susanta Roychoudhury

**Affiliations:** 1 Amity Institute of Molecular Medicine and Stem Cell Research (AIMMSCR), Amity University, Sector-125, Noida, Uttar Pradesh, 201313 India; 2 Division of Research, Saroj Gupta Cancer Centre and Research Institute, Kolkata, 700063 India; 3 Molecular Genetics Division, CSIR-Indian Institute of Chemical Biology, Kolkata, India

**Keywords:** p53, Amyloid, Mutations, Cancers, Signaling pathways, Targeted therapies, CRISPR-Cas9

## Abstract

The tumor suppressor p53 when undergoes amyloid formation confers several gain-of-function (GOF) activities that affect molecular pathways crucial for tumorigenesis and progression like some of the p53 mutants. Even after successful cancer treatment, metastasis and recurrence can result in poor survival rates. The major cause of recurrence is mainly the remnant cancer cells with stem cell-like properties, which are resistant to any chemotherapy treatment. Several studies have demonstrated the role of p53 mutants in exacerbating cancer stemness properties and epithelial-mesenchymal transition in these remnant cancer cells. Analyzing the amyloid/mutant p53-mediated signaling pathways that trigger metastasis, relapse or chemoresistance may be helpful for the development of novel or improved individualized treatment plans. In this review, we discuss the changes in the metabolic pathways such as mevalonate pathway and different signaling pathways such as TGF-β, PI3K/AKT/mTOR, NF-κB and Wnt due to p53 amyloid formation, or mutation. In addition to this, we have discussed the role of the regulatory microRNAs and lncRNAs linked with the mutant or amyloid p53 in human malignancies. Such changes promote tumor spread, potential recurrence, and stemness. Importantly, this review discusses the cancer therapies that target either mutant or amyloid p53, restore wild-type functions, and exploit the synthetic lethal interactions with mutant p53.

## Introduction

**Fig. 1:**
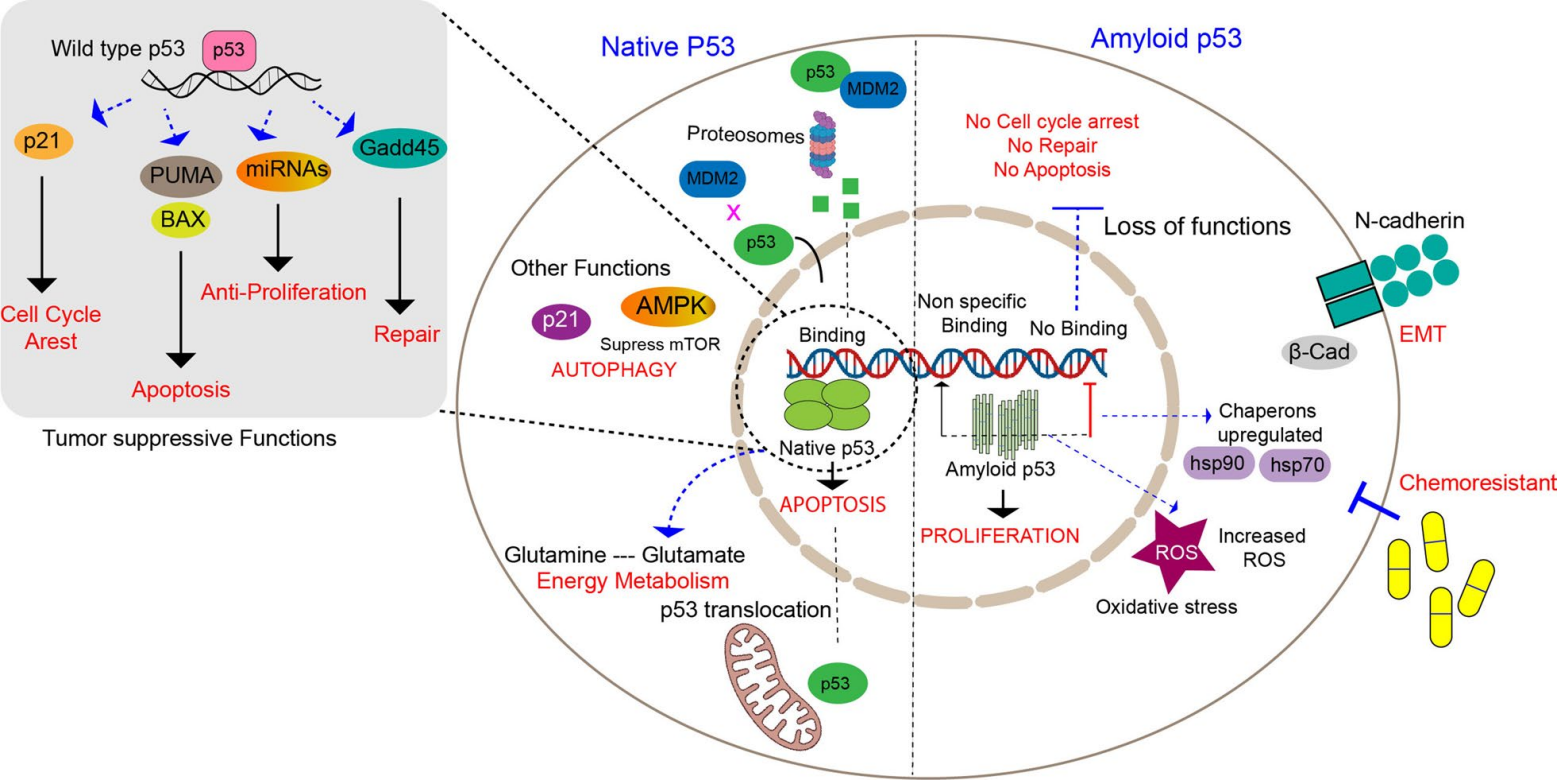
Schematic showing the fate of wildtype p53 versus amyloid p53. The cellular consequences due to p53 amyloid formation when compared to wildtype p53 of cells harboring p53 aggregates. Native p53 is a tetrameric transcription factor that regulates several genes by binding to the p53-specific response element (RE) to control the apoptotic, cell cycle arrest, and DNA repair pathways. To maintain cellular homeostasis, native p53 controls cell cycle and proliferation as well. On the other hand, via altering cellular networks primarily involved in the cell cycle, DNA repair, and cell proliferation, amyloid p53 conveys the gain of oncogenic properties to the cells. Amyloid formation causes a significant upregulation of pathways involved in the unfolded protein response, chaperones (Hsp70, Hsp90), and proteasomal machinery. Genes and proteins involved in apoptosis and senescence pathways are downregulated because of p53 amyloid accumulation, making the cell susceptible to oncogenic transformation. Additionally, because of the production of p53 amyloid, genes involved in cellular signaling that promote cell cycle and proliferation (CDKs, MAPK, ERK, CDCs, and Ras) are elevated concurrently. These pro-oncogenic genes give cells harboring p53 aggregates benefits in growth, migration, and survival. Furthermore, EMT, stemness, chemoresistance, and metastasis are caused by the overexpression of proteins in translational and metabolic pathways

The function of tumor suppressor p53 is well-highlighted in human cancer, where it is mostly present either in mutated or amyloid/aggregated form [1, 2]. Under normal conditions, p53 is inactivated by MDM2 (murine double minute 2), a negative regulator that causes proteasomal degradation of p53 [3, 4]. Phosphorylation of p53 caused by diverse cellular stresses can reduce its binding affinity to MDM2, which results in its activation [4]. The p53 protein subsequently forms a homo-tetramer that binds to specific p53 response elements in the genomic DNA, where it acts as a transcriptional regulator of its downstream genes, regulating the cell cycle, apoptosis, DNA repair, and several other vital functions to maintain the genome’s integrity [4, 5] (Fig. 1). Control of p53 activity is achieved by post-translational modifications, such as phosphorylation, acetylation, and ubiquitination, which influence p53 binding to the DNA and also allow interaction with other proteins thereby affecting p53 transcriptional function [6]. On the other hand, mutant p53 exhibits the loss of its obligatory transcriptional activities, thereby gaining new functions [6–10]. Previous studies suggest that in tumor cells, a non-functional p53 is often present in an aberrant, misfolded, and inactive conformation [11, 12]. Amyloid formation begins with the unfolding of p53 structural folds, followed by the formation of β-strand containing structures [12]. Recent studies on the aggregation of p53 showed its internalization into cells, its seeding capacity, and cell-to-cell transmission in a prion-like fashion [13]. Interestingly, not only wildtype p53 but also mutant p53 can also form amyloid, thereby leading to tumorigenesis. Mutant p53 can intensify the amyloid-forming kinetics by destabilizing the protein fold, as observed with R175H, a well-studied hotspot mutation [12]. Due to its amyloid conformation, p53 acquires a dual role in cancer initiation and progression through the loss of tumor suppressive functions and gain of tumorigenic properties [11–14] (Fig. 1).

A non-functional mutant p53 is known to interact with several transcriptional factors (TFs) to influence gene expression, leading the tumor cell towards metastasis [8, 9, 15, 16]. Previous reports have provided evidence linking the loss or gain of p53 functions to the induction of epithelial-mesenchymal transition (EMT) as well as the acquisition of cancer stemness properties in different tumor cell lines as a result of a gain of function phenotype [10, 15–18]. In this review, we provide a consolidated report, deciphering the role of mutant and amyloid p53-mediated mechanisms in metastasis via EMT transition or relapse due to cancer stemness. Interestingly, we highlighted the missing link between p53 amyloid formation and cancer initiation, progression, and metastasis (Fig. 1). Further, we have explained all the important signaling pathways and discussed how these signaling molecules could be altered for precision medicine. Lastly, we explore the strategies for functional reactivation of mutant and amyloid as a potential therapy.

## Amyloid/mutant p53 gain-of-function

The transition of p53 from protector to tormentor can result from either mutation or amyloid formation of both wildtype and mutant forms [7, 10–13, 13, 18–24]. Studies on aggregated p53 reveal the inability of the oligomeric and fibrillar forms to bind with the DNA using in vitro models [11]. In the absence of a functional nuclear p53, all transcriptional activity necessary for maintaining both tumor-suppressing and normal cellular functions, such as cell cycle arrest and apoptosis is disrupted. Heat shock proteins (Hsp90 and Hsp70) are overexpressed because of aggregated p53 (R175H mutant), which increases tumorigenic cell proliferation while inhibiting apoptotic mechanisms [25]. The p53 amyloids are reported to be highly stable and infectious in nature, can display prion-like behavior, and exhibit cell-to-cell transmission, rendering the native p53 non-functional [13]. p53 oligomers and fibrils present in several human tumor biopsies were also observed in the brains of the patients with Alzheimer’s but not in the control brain, suggesting their devastating effect beyond cancer [26]. Recently, it was reported that p53 amyloids had the potential to establish a tumor xenograft in immunocompromised mice [13]. Moreover, several other studies have also supported the contributions of mutant and amyloid p53 GOF toward cell proliferation, invasion, and metastasis [18, 27–29].

The *TP53* gene mutations can be mainly categorized into two types, such as contact mutants (R248Q, R273H, and R248W) and conformational mutants (G245S, R175H, R249S, and R282H) [30]. Both the mutants type are well known to affect the DNA binding domain (DBD) [31]. The contact mutants exhibit changes in the amino acids directly responsible for binding their response element, whereas structural mutants undergo conformational changes resulting in misfolded proteins, which may or may not be able to enter the nucleus [32]. Both the mutant and amyloid forms exhibit loss of function and gain-of-function phenotypes [10, 11, 13, 19, 20, 27, 28]. Studies have shown the ability of many mutant p53 to bind and inactivate p53 family proteins such as p63 and p73 [33]. This binding of mutant p53 with p63 and p73 is associated with chemoresistance, proliferation, and metastasis of the cancer cells [34]. R248Q, one of the hotspot mutants of p53 promote cancer survival upon glutamine starvation [35] since for most of the cancer cells, glutamine is required for proliferation [33]. When compared to patients with p53 null tumors, the patients carrying mutant p53 displayed a worse prognosis, a poor response to treatment, and a faster tumor recurrence [28, 36]. Similar to mutant p53, cells with the amyloid form of p53 demonstrate enhanced motility and increased adhesion to collagen with higher focal adhesion complex formation in wound-healing assays [12, 14]. Additionally, upregulation of the oncogenes such as MAPK1 and CCND2 further displays the oncogenic potential of the amyloid form [12].

### Potential of mutant/amyloid p53-driven cancer stemness

**Fig. 2:**
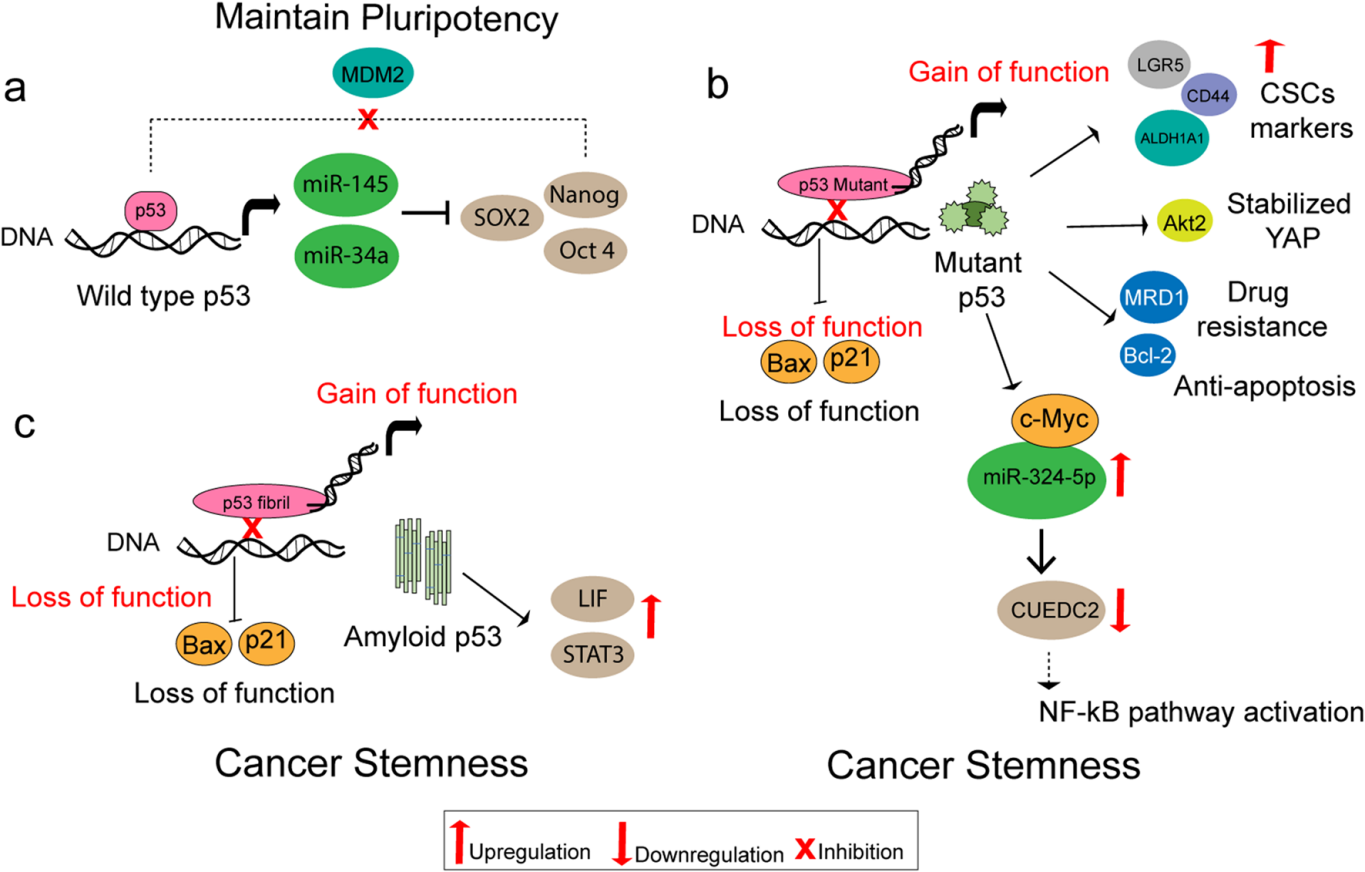
Schematic showing p53 role in cancer stemness. a. Wildtype p53 can modulate the expression of stem cell transcription factors to control pluripotency. Nanog can suppress p53 activity by activating Mdm2 to promote pluripotency. b. Mutant p53 can activate the CSC markers such as ALDHA1, CD44, and LGR5 by binding to their promoters to promote stemness. By upregulating the anti-apoptotic genes Bcl-2 and the multidrug resistance gene MDR1, mutant p53 can encourage the characteristic CSC phenotype of increased drug resistance and prolonged survival. Mutant p53 regulates c-Myc and can increase the expression of miR-324-5p resulting in the downregulation of CUEDC2, a miR-324-5p downstream target gene, which activates NF-kB pathway exhibiting cancer stemness features. c. Amyloid p53 can upregulate LIF and STAT3 probably contributing to the cancer stemness phenotype

p53 is known to balance self-renewal and differentiation to sustain a pool of stem cells for healthy development and the preservation of tissue homeostasis [9]. p53 is negatively regulated by E3 ubiquitin ligases HDM2 and TRIM24, thereby maintaining low p53 levels in human embryonic stem cells (hESC) [9]. Acetyltransferases CBP/p300 can acetylate p53 leading to the dissociation of TRIM24 and HDM2 [9]. This results in p53 activation that can further activate downstream partners such as miR-34a, and miR-145 [37] (Fig. 2). Activation of miR-34a and miR-145 can maintain pluripotency by inhibiting the stem cell markers such as Lin28a, Oct4, Klf4, and Sox2 genes (Fig. 2). On the other hand, it was observed that homozygous deletion of p53 can develop a stemness phenotype in pancreatic acinar cells showing elevated expression of cancer stem cell (CSC) markers and stem cell regulators such as c-Myc, SOX9, Klf4 along with several other genes [9]. Therefore, the loss of p53 can also result in the development of stem cell-like characteristics that enhance tumor growth.

In a recent report, the normal breast cell line MCF-10A was treated with the in vitro aggregated p53 fibril containing the. DNA binding domain region along with an untreated control and were cultured up to 5^th^ generation [13]. When the extracted RNA was subjected to microarray analysis it was observed that *STAT3* was upregulated in the T5 generation suggesting a role of amyloid p53 in maintaining the stem cell phenotype via *STAT3* upregulation [13]. Interestingly, it was also found that Leukemia Inhibitory Factor (LIF) was significantly overexpressed in both the T1 (first generation) and T5 generation of MCF-10A cells carrying amyloid p53 [13]. LIF along with its receptor (LIFR) form a part of the interleukin-6 (IL-6) cytokine family LIF/LIFR signaling pathway, which plays an important role in tumor progression, stemness, and resistance to therapy [38]. Many solid cancers have been reported to overexpress LIF [39]. Upregulation of LIF can in turn upregulate the JAK-STAT pathway through phosphorylation of STAT3 (Fig. 2). Based on the above observations, it can be stated that amyloid p53 can drive the cell towards tumorigenesis and stemness as a gain-of-function event via the LIF/STAT3 pathway. However, additional data are required to validate the mechanism involving p53 amyloid and cancer stemness.

Several p53 mutants can display a GOF phenotype and cause stemness like features in tumor cells via targeting various pathways (Fig. 2). Mutant p53 can either directly activate several CSC markers such as ALDHA1, CD44, and LGR5 to promote stemness [40] or can promote stemness indirectly by activating PI3K/AKT2-mediated integrin or growth factor receptor cycling, as reported in glioblastoma and breast cancer cells [9]. AKT2 phosphorylates WASP-interacting protein by AKT2 thereby stabilizing YAP/TAZ transcriptional factors to support cancer stem cell survival and phenotype (Fig. 2). Another path that the mutant p53 takes towards stemness is either by enhancing the expression of MDR1, a multidrug resistance gene, or upregulating Bcl-2 for enhancing drug resistance and survival [9]. A recent report demonstrated that an oncogenic transcription factor, c-Myc, can upregulate miR-324-5p expression in the presence of GOF mutant p53 in cancer cells [41]. This causes CUEDC2, a downstream target gene of miR-324-5p to be downregulated resulting in NF-kB activation, which can confer cancer stemness traits [41].

### Potential role of mutant/amyloid p53 in EMT transition

Epithelial-mesenchymal transition (EMT) has been studied in detail and is implicated in carcinogenesis and triggering metastatic properties by enhancing proliferation, migration, invasion, and resistance to apoptotic stimuli [42]. Amyloid p53 is shown to play a vital role in EMT [43]. MCF10A cells showed three-dimensional spheroid formation when cultured in Matrigel after inducing p53 amyloid formation [13]. The diameter of spheroids gradually increased over 10 days, indicating the proliferation and migration of cells containing amyloid p53 [13]. Furthermore, the spheroids showed an increase in the expression of the EMT markers (β-catenin, Vimentin, Slug, and N-cadherin) [13]. This suggests that cells with amyloid p53, like p53 mutants, can stimulate EMT-like gain of function effect. In addition to oncogenic transformation, the aggregated p53 induces cytotoxic effects on some cells which may result in the release of the p53 aggregates into the extracellular environment [10]. Altogether, the above fact revealed that p53 aggregates can be transmitted between two cells [12, 43]. In this context, recent reports have also established the prion-like transfer of p53 aggregates from mother to daughter cells [13, 14, 26]. This transfer of p53 aggregates gives an oncogenic advantage to the cell leading to increased survival. In all these processes, cellular toxicity was not observed; rather, it gave survival benefits to the cell by overcoming apoptosis as well as the senescence mechanisms [12]. These data hint at the dissemination tendency of these aggregates. Reports have shown that the aggregated p53 can be internalized into the cells by micropinocytosis. However, the detailed mechanism of transmission is yet to be investigated in detail. Based on the above observations and the available literature for the transmission of prion-like aggregates [44], numerous possible mechanisms of transmission between neighboring cells can be hypothesized and explored in the future.

Several p53 mutations, such as R248W, R175H, and R272H were found to down-regulate the expression of E-cadherin and up-regulate the expression of β-catenin and laminin V in MCF10A [45]. Interestingly, all these hotspot mutations were shown to form amyloid under in vitro conditions [12, 29]. In colon cancer, cells harbouring mutant p53 can trigger EMT by expressing the stem cell markers c-Myc, CD44v6/CD133, and Zeb1 [46]. In a similar study, several of the mutant p53 (GOF: R175H, R273H, D281G, and V143A) in colorectal cancer cells can simultaneously boost chemoresistance and cause EMT via the expression of EMT-TFs Snail and Slug [46]. Interestingly, in a recent study, knockdown of the endogenous expression of wildtype p53 also suggested its role in EMT. These effects may be distinct to cell type and context-dependent, as indicated by a recent report where silencing of the WT p53 gene in HepG2 cells increased its angiogenic ability but not the migratory ability when co-cultured with endothelial cells. However, the p53 null cell line did not exhibit enhanced angiogenic potential by itself but showed increased migration when co-cultured with endothelial cells [47]. One of the p53 targets, PTK2 (focal adhesion kinase) was observed to be overexpressed in invasive breast and colon cancers. PTK2 is responsible for promoting cell invasion by integrin-mediated signaling [48], which otherwise is likely to be downregulated by p53 to suppress metastasis [48]. Therefore, loss of p53 functions due to amyloid formation or mutation might result in the upregulation of such genes involved in metastasis, resulting in aggressive disease progression.

## Altered metabolic and signaling pathways due to p53 mutation/amyloid formation

For a normal cell to become cancerous and eventually develop a metastatic phenotype, it often needs to go through several changes [46]. Cancer initiation and progression have been linked to several specific changes in gene expression patterns. p53 amyloid formation also contributes to such a cascade of changes in the cellular pathways [49]. We further summarize the immediate effect of p53 amyloid formation and/or p53 mutations and their eventual consequences on the major signaling pathways of the cell.

### Mevalonate metabolic pathway

**Fig. 3:**
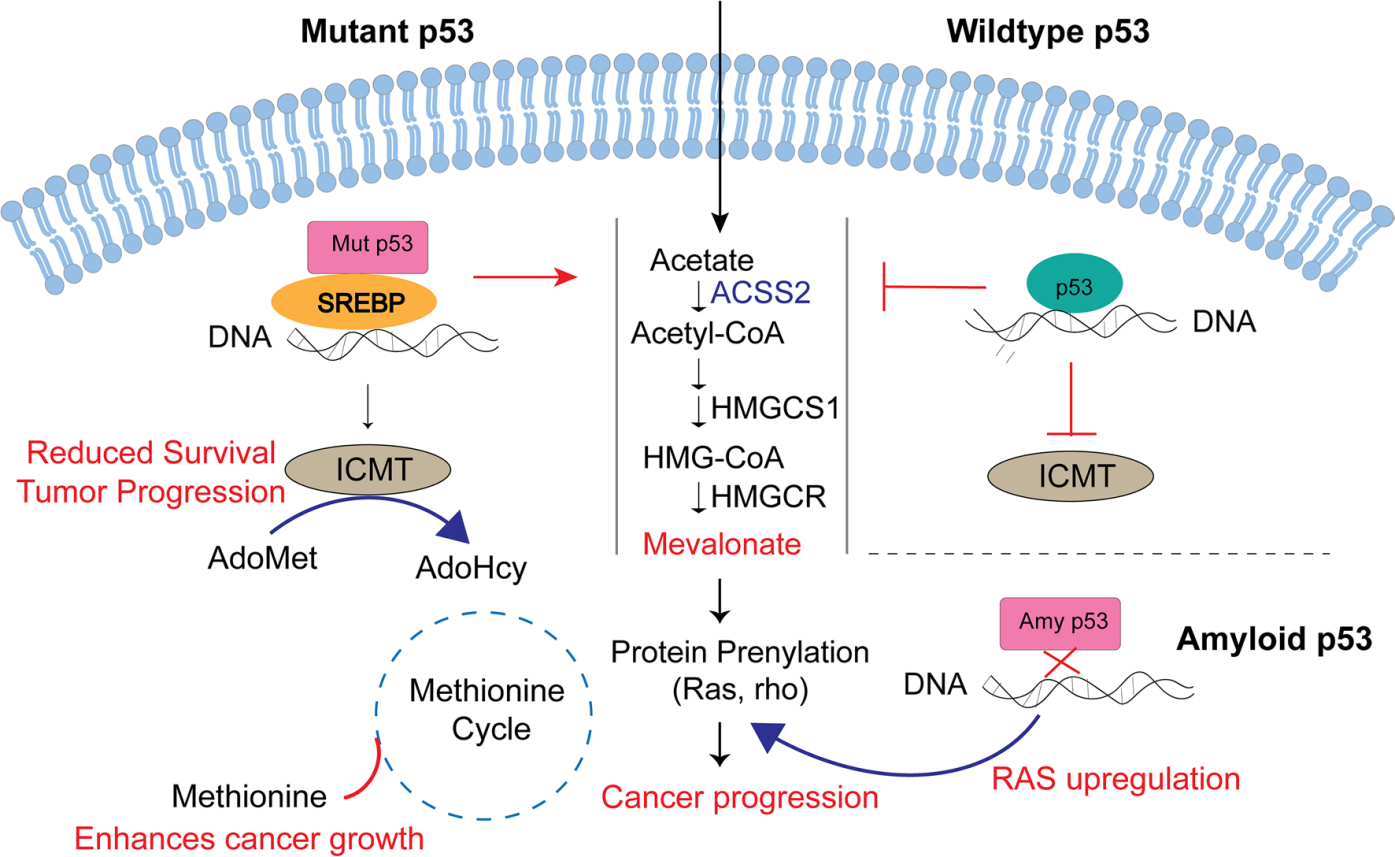
Role of p53 in regulating the mevalonate pathway. Acetate can be converted to acetyl-CoA, which can then enter the mevalonate pathway and be further converted into 3-hydroxy-3-methylglutaryl CoA in a two-step synthesis (HMG-CoA). Then, 3-hydroxy-3-methylglutaryl-CoA reductase (HMGCR) reduces HMG-CoA to provide mevalonate. Then, mevalonate can control the enzymatic processes that lead to protein prenylation. Sterol regulatory element binding proteins (SREBPs) can interact with p53 mutants to promote the expression of mevalonate pathway genes. On the other hand, as a result of the transcriptional upregulation of ATP binding cassette subfamily A member 1, wild type p53 (WT p53) represses the genes involved in the mevalonate pathway by preventing SREBP-2 from maturing (ABCA1). Additionally, while WT p53 acts as a transcriptional repressor, mutant p53 can activate the isoprenylcysteine carboxyl methyltransferase (ICMT) gene. The final stage of the protein prenylation pathway, protein carboxymethylation, is catalyzed by ICMT. The rate-limiting reaction, which is carried out by the enzyme methionine adenosyl transferase, converts the important amino acid methionine into S-adenosyl methionine (SAM), the methyl donor in this reaction (MAT). S-adenosyl homocysteine (SAH), which is needed in the methionine cycle to replenish methionine, is created when SAM is converted. Ras upregulation by amyloid p53 might activate several other signaling networks controlling differentiation, survival, and cell proliferation

The mevalonate (MVA) pathway is a vital metabolic system that forms acetyl-CoA to create sterols and isoprenoids, both of which are important for tumor growth and progression [50]. The metabolism of cancer cells is reprogrammed to provide energy and the fundamental building blocks required for their abnormal survival and proliferation. Such reprogramming changes the expression of important metabolic enzymes of signaling pathways including the mevalonate pathway (Fig. 3). Wildtype p53 can block the activation of the master regulator of mevalonate pathway SREBP-2 by transcriptionally inducing the ABCA1 cholesterol transporter gene to suppress tumorigenesis (Fig. 3). Mutant p53 on the other hand can enhance the expression of mevalonate pathway enzymes by direct interaction with SREBP2 thereby enhancing the expression of mevalonate pathway genes as well as activating oncogenic proteins, Ras, Yap, Rho [50]. Amyloid p53 was also observed to upregulate KRAS [43] thereby activating several signaling networks controlling differentiation, survival, and cell proliferation [43]. There was overlap in nine pathways, including the p53 pathway and Kras signaling, on comparison between pathways affected by amyloid versus mutant p53 [43]. p53 amyloids behave similarly to aggressive oncogenic p53 mutants, as seen by the overlapped processes caused by p53 amyloids and p53 mutants, which both lead to gain-of-function features [43]. However, detailed studies on the regulation of the mevalonate pathway by amyloid p53 are unexplored.

### TGF-β signaling pathway

The transforming growth factor-β (TGF-β) protein family includes TGF-β, activins, and bone morphogenic proteins (BMPs). The TGF-β family controls several critical cellular functions including apoptosis, proliferation, differentiation, EMT, and migration. TGF-β is known to exhibit a dual function as a tumor suppressor and a tumor promoter in cancer [51]. Several studies have found a link between p53 and TGF-β signaling [45, 46, 52]. The metastatic properties of TGF-β can be acquired by the actions of Ras and mutant p53 [53]. Additionally, RAS is frequently altered in cancer, which causes changes in cellular adhesion and motility, which enable cancer cells to become more invasive and metastatic. Studies have shown that Ras-activated mutant-p53 and TGF-β can inhibit the transcriptional activity of p53 family member p63. p63 can protect epithelial stem cells from apoptosis and coordinate their differentiation [48]. Therefore, the inhibition of p63 due to the binding of a protein complex involving mutant-p53 and TGF-β activates Smads, disabling metastatic protection [54]. This further inhibits the known targets of p63, Sharp-1, and Cyclin G2, which can suppress tumor cell migration and metastasis. In a separate study on hepatocellular carcinoma (HCC), knockout of p53 resulted in TGF-β1-induced changes of critical EMT markers [55]. p53 is frequently mutated in HCC exhibiting loss of function or gain of new function [48]. The absence of p53 could elevate the metastatic potential of the tumors by invading the lungs and bile ducts [55]. The expression of other TGF-β-induced genes such as p21, PAI-1, and MMP2 also requires the interaction of p53 and Smads. TGF-β can induce p53/Smads complex formation, thereby recruiting histone acetyltransferase CREB-binding protein (CBP) to the PAI-1 promoter, resulting in the acetylation of histone to activate PAI-1 transcription by relaxing the chromatin structure [54]. A hotspot p53 mutant R175H can affect TGF-β signaling pathway as observed in human H1299 lung cancer cells. R175H suppresses TGFBR2 expression, resulting in the reduction of SMAD2/3 phosphorylation [56]. This results in the inhibition of SMAD translocation to the nucleus [56]. Therefore, mutant p53 acquires the gain of function effect by the disruption of the balance of SMAD transcriptional activity [57]. Although the R175H mutant is reported to undergo amyloid formation [12], the crosstalk of p53 amyloid with the TGF-β signaling pathway has not been explored yet.

### PI3K/AKT/mTOR signaling pathways

The mammalian target of rapamycin (mTOR) is a serine/threonine molecule downstream of the phosphatidylinositol 3-kinase (PI3K) signaling pathway that controls cell growth and is responsible for the regulation of proliferation, autophagy, growth, survival, mobility, and angiogenesis. The mTOR complex consists of mTORC1 and mTORC2, each with distinct functions. mTORC1 controls cell growth in response to nutrient availability and growth factors, and mTORC2 can mediate cell proliferation and cell survival. AKT (serine-threonine protein kinase family) is phosphorylated, activated, and localized in the plasma membrane by PI3K [58]. AKT can in turn activate genes such as CREB (cAMP-response element binding protein) [58], suppress p27 [59], and activate mTOR [59], thereby reducing apoptosis and enhancing proliferation [58]. Anti-apoptotic genes (AKT1, SEPT4, and BAD) were elevated in the global gene expression analysis of MCF10A cells harboring p53 amyloids at two separate generations, the initial (T1) and later (T5) generation [43]. Previous reports have suggested colon cancer cells driven by RAS mutations resulted in the upregulation of AKT-1, which in turn regulated the autophagy mechanism [60]. Such observations suggest that p53 amyloid may play a vital role in tumor progression by regulating autophagy or modulating the PI3K/AKT/mTOR cell signaling pathway, although a detailed mechanism is not yet explored. In osteosarcoma cells, p53 is a potent inhibitor of mTOR. p53 expression was able to reduce the motility and invasion ability of these cells, suggesting that the mTOR pathway also participates in the process of tumor invasion and metastasis. Amyloid forming mutant p53 (R175H/R273H) via the transcriptional repression of the downstream p53 autophagy responsive genes, such as ATG12, BECN1, TSC2, DRAM1, SESN1/2, and P-AMPK can suppress the formation of autophagic vesicles as well as their fusion with lysosomes, thereby leading to autophagy blockage [61, 62]. Several of the p53 mutants can suppress Beclin-1 expression in autophagy. Furthermore, certain cancer-associated p53 mutations can interact with other TFs capable of suppressing autophagy indirectly by activating various growth factor receptors, such as TGFBR, EGFR, and IGFR [7], resulting in persistent active PI3K/Akt/mTOR signaling and repression of autophagy. Mutant p53 can promote proliferation, invasion, and metastatic potential by altering the endosomal pathway, which results in the recycling of receptors and integrins. It has been demonstrated that overexpressing the mutant p53 causes an increase in the translocation of EGFR and α5β1 integrin on the surface of cell membranes. RCP (Rab-coupling protein) interaction is required for this translocation [34, 48]. Thus, mutant p53 activates many intracellular pathways involved in the control of endosomal pathways, such as the PI3K/AKT or MAPK cell signaling pathways.

### WNT signaling pathway

Wnt signaling is another key pathway that is associated with carcinogenesis and can regulate stemness. The Wnt pathway administers cell proliferation and cell fate in multiple tissues in several organisms [63]. However, mutations can lead to aberrant activation of Wnt signaling, providing an oncogenic stimulus to cells [64]. In breast cancer, the loss of TP53 could elicit the secretion of several Wnt ligands, such as Wnt1, Wnt6, and Wnt7a [64, 65]. These Wnts bind to primary receptors on the surface of tumor-associated macrophages, stimulating them to produce proinflammatory cytokines like IL-1β. IL-1β stimulates the expression of another proinflammatory cytokine, IL-17, in Gamma delta T cells (γδ T), resulting in the expansion of the neutrophils due to the granulocyte colony-stimulating factor (G-CSF) [64, 66]. Inducible nitric oxide synthase (iNOS), which is produced by phenotypically changed neutrophils, reduces the function of antitumor CD8 + T lymphocytes, causes systemic inflammation, and promotes the spread of breast cancer [66].

Studies have shown that the mouse embryonic stem cell differentiation program preferentially targets the Wnt signaling pathway. [67]. Crosstalk between p53 and Wnt signaling pathway can result in stem cell acquisition. Wip1, one of the p53 downstream phosphatase was reported to be associated with p53-dependent apoptosis of stem cells in the mouse intestine [68]. In Wip1 deficient mice, low levels of Wip1 could reduce the threshold of p53-dependent apoptosis of the stem cells. However, Wip1 deficiency does not affect the localization and nuclear levels of β-catenin, contributing to the up-regulation of c-Myc and Cyclin D1. The role of p53 in controlling EMT is also demonstrated by the p53-miR-34-Wnt network, regulating the stem cell phenotype and tumor progression. One of p53's direct downstream targets, miR34 is known to interact with the Wnt and EMT genes -catenin, AXIN2, and Snail. p53 loss due to miR-34 activated the Wnt pathway, which further induces the transformation of EMT [69]. Therefore, p53 plays a vital function in controlling EMT. A similar observation was seen in acute myeloid leukemia (AML) where Wnt signaling pathway genes were shown to be upregulated in cells with wild-type p53 as compared to mutant p53 [70]. Amyloid p53 can upregulate Wnt-11 in MCF10A cells [43]. Enhanced Wnt-11 function was observed to improve the survival of the CHO cell [60]. The Wnt signaling pathway was observed to be down-regulated in response to the activation of Wnt-11 activity, suggesting a role for Wnt-11 in cell viability [60]. Thus, p53 amyloid can regulate the Wnt signaling pathway via Wnt-11 to enhance cell survival. However, a detailed study regarding the p53 mutant and modulation of the Wnt signaling pathway is not reported.

### NF-κB signaling pathway

Nuclear factor kappa B (NF-κB) has an essential regulatory function in immune responses, inflammation, cell proliferation, and apoptosis [71]. NF-κB is bound to its inhibitory protein, IκB when inactive. On receiving stimulus from cytokines or growth factors, IκB is phosphorylated, and targeted for ubiquitination followed by degradation by the proteasome. NF-κB, now free from its inhibitor, can translocate to the nucleus and induce expression of immunoreceptors, other transcriptional factors, cytokines etc. [71]. In mammalian cells, five distinct NF-κB subunits are present, namely, NF-κB2(p100/p52), NF-κB1(p105/p50), RelA(p65), RelB, and c-Rel containing a highly conserved amino-terminal DNA-binding and dimerization domain [72]. p53 could induce p21 and indirectly stimulate the activity of NF-κB and other transcription factors utilizing p300 and CBP [73]. WT and mutant p53 were found to have opposing roles in head and neck cancer cells. A study reported that silencing of the p65 subunit of the NF-kB complex leads to activation of EMT in cells with mutant p53, while overexpression of NF-kB activates EMT in cells with the WT p53 gene [71, 74]. NF-κB stimulation is also associated with resistance to programmed cell death. NF- κB and p53 are reported to have the ability to inhibit each other [73]. The cell fate towards apoptosis will depend on the cross-talk between NF-κB and p53 which is further determined by the nature of the stimuli, however, mutant p53 is shown to not interfere with the NF-κB functioning [73]. Instead, mutant p53 can protract the TNF-α-induced NF-κB activation in the colorectal cancer cells as well in the organoid models [75]. The direct role of p53 amyloid in modulating the NF-κB signaling pathway is not yet explored.

## Effect of mutant/amyloid p53 on non-coding RNAs

### miRNA modulations

MicroRNAs (miRNAs) are small noncoding RNAs that can regulate the expression by binding to the 3′-UTR of RNA molecules [76]. Several of these miRNAs are reported to be associated with the p53 signaling pathways. p53 was observed to suppress the expression of two transcription factors and EMT markers, ZEB1 and ZEB2 which are known to promote EMT [77]. Profiling of HCC cell lines has revealed that p53 can up-regulate several miRNAs, such as miR-200 and miR-192 family members. p53 can bind to the promoter of these miRNAs thereby repressing the expression of ZEB1/2, for driving EMT phenotype in human cancers [77]. On the other hand, mutant p53 is observed to promote EMT by inhibiting the transcription of miR-130b in endometrial cancer [31]. Moreover, miR-130b is known to be a negative regulator of one of the EMT markers, ZEB1 [16].

Another well-studied mRNA is miR-145, which is involved in regulating EMT and stemness [78]. miR-145 is known to be a direct target of p53 [76]. Wildtype p53 enhances miR-145 expression in prostate cancer cells, resulting in inhibition of migration, invasion, and EMT phenotype. Further, miR-145 is also shown to repress the stemness of prostate cancer cells by suppressing the expression of c-Myc, CD44, Oct4, and Klf4 genes [76]. Therefore, the loss of the wildtype p53 most likely promotes bone metastasis of prostate cancer cells partially by repressing miR-145 to elevate the EMT and stemness of cancer cells. In a separate study mutant p53 was able to induce the secretion of miR-1246-enriched exosomes that could function to promote cancer progression and metastasis in colon cancer cells [79]. The uptake of these exosomes by macrophages resulted in miR-1246-dependent reprogramming by the secretion of tumor-supportive factors [80]. p53 can also directly induce the transcriptional activation of miR-200c, reported in regulating the EMT process through inhibition of transcriptional suppressors of an epithelial marker, E-cadherin [81]. miR-200c can target and suppress the E-cadherin transcriptional suppressor ZEB1/2 [82], thereby regulating the EMT process [80].

### Regulation of specific long non-coding RNAs (lncRNAs)

LncRNAs are generally more than 200 nucleotides and lack protein-coding potential [74, 75]. Recent studies have indicated that these lncRNAs can play a role in tumor initiation, progression, as well as metastasis, and are novel molecular biomarkers for the diagnosis and prognosis of cancer patients [83, 84]. In pancreatic ductal adenocarcinoma (PDAC), p53 is shown to regulate gene expression by inducing enhancer RNAs and large intergenic noncoding RNAs (lncRNAs) [85]. Neat1, for instance, was recently discovered to be a p53-induced lincRNA, and it has been demonstrated that its absence leads to PDAC malignancy via broad alterations in gene expression [85]. Some lncRNAs are known to regulate p53 indirectly via MDM2 [80]. LncRNA-PRAL can decrease the p53-MDM2 interactions, thereby inhibiting MDM2-induced p53 degradation. Overexpression of lncRNA-PRAL can inhibit HCC growth by inducing apoptosis through p53 [86]. A similar observation is seen in the case of pancreatic cancer, where lncRNA, CF129 can bind to mutant p53 and the E3 ligase, MKRINI resulting in the degradation of mutant p53 via ubiquitination [87]. Several of lncRNAs play important roles in cancer progression via p53 suppression or degradation. Consistent with that, in the case of colorectal cancer, PURPL was identified as a p53-responsive LncRNAs [88]. PURPL can bind the RNA-binding protein HuR thereby forming a stable complex with MYBBP1A that can function to destabilize p53. Thus, PURPL is a p53 transcriptional target that modulates basal p53 levels [88]. Additionally, the lncRNA MALAT1 can decrease the acetylation process of p53 with help of a protein deacetylase SIRT1. MALAT1 is reported to be highly expressed in non-small cell lung cancer and is indicative of poor prognosis. When overexpressed it can suppress the transcription of p53 target genes responsible for proliferation by binding only to mutant p53 [88].

## Mutant/Amyloid p53 contribution towards chemoresistance

Chemoresistance is one of the gain-of-functions brought about by mutant p53 in cancer cells. Mutant p53 can regulate several pathways which can directly promote resistance to chemotherapeutic drugs including cisplatin, alkylating agents (temozolomide), antimetabolites (gemcitabine), anthracyclines, (doxorubicin), antiestrogens (tamoxifen) and EGFR-inhibitors (cetuximab) [89]. Several studies on different p53 mutations have well-documented the fact that mutant p53 can confer chemoresistance in cancer cells [90]. Under these studies, pieces of evidence have also been provided for p53 amyloids. Recently, in viability assays, it was observed that MCF 10A cells containing p53 amyloids displayed significantly high EC50 values with drugs such as cisplatin, doxorubicin, and paclitaxel than cells with native p53 protein [13]. The role of amyloid-like mutant p53 oligomers was also seen in the chemoresistance phenotype of malignant and invasive brain tumors [21]. In the case of a subset of high-grade serous ovarian carcinoma patients with p53 aggregation, poor chemo-response was observed again, suggesting p53 aggregation as a new marker for chemoresistance [91]. Further, inhibiting p53 aggregation was able to reactivate the p53 pro-apoptotic function [91]. Based on these observations, it appears that p53 amyloid formation can induce drug resistance in tumor cells like mutant p53. However, the detailed mechanism is still under investigation.

## Relevance of p53 amyloid in the clonal evolution of cancer

**Fig. 4:**
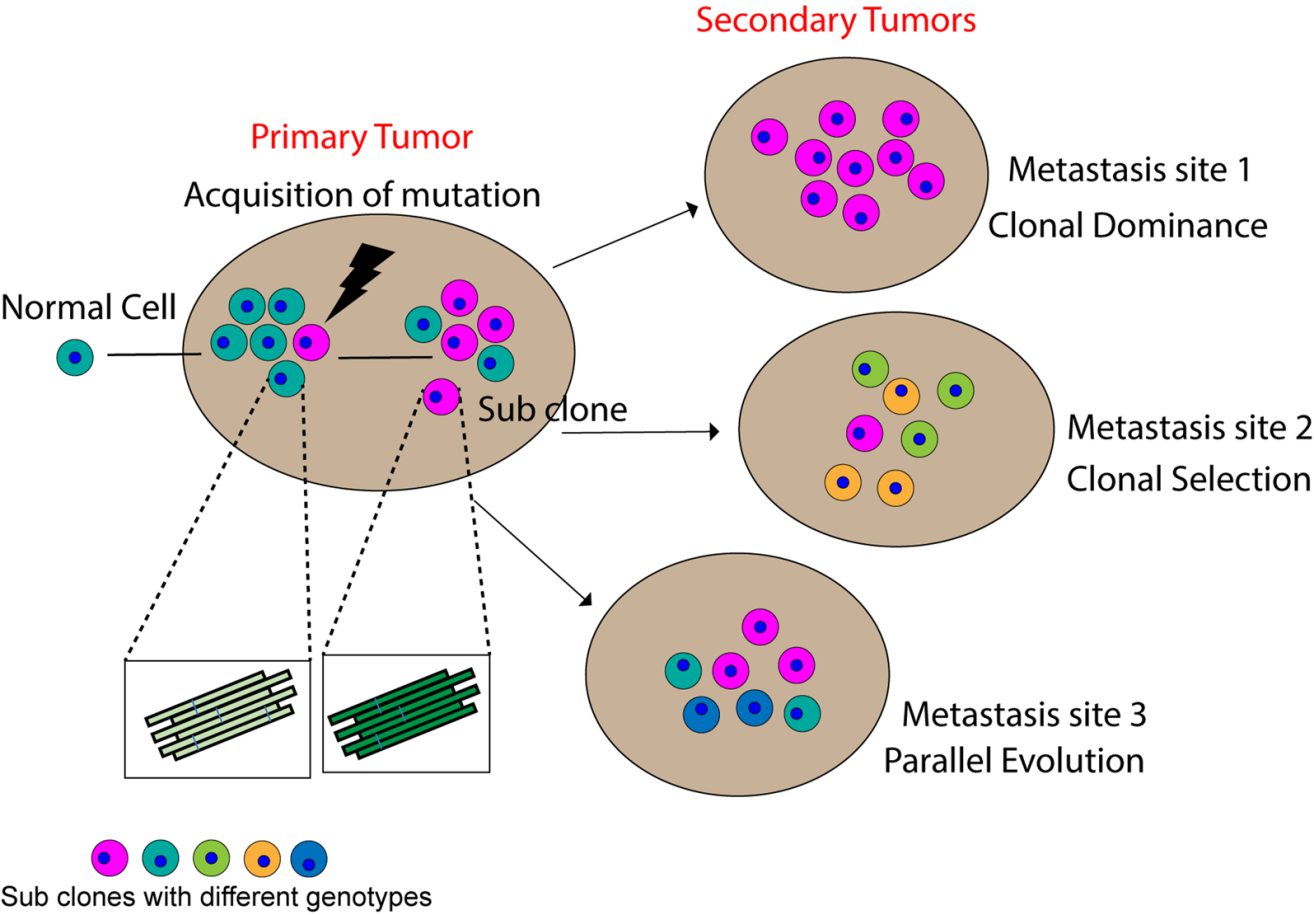
Schematic showing clonal evolution in cancer and the possible role of p53 amyloid. According to the clonal dominance hypothesis, metastatic subclones inside the main tumour may outgrow and take control of the tumour bulk. According to the clonal selection model of metastasis, the subpopulations of cell populations that are capable of spreading are the ones that do so. As per the parallel evolution hypothesis, metastasis develops early in the course of a tumour and is not dependent on the presence of tumour cells in the primary site. Due to the various mutations, the subclones have different genotypes. We hypothesize that these mutations acquired by p53 gene results in amyloid formation with varied phenotypes leading to clonal evolution

One of the crucial phenomena for cancer development, progression, and metastasis is clonal evolution (Fig. 4). Cancer is known as an evolutionary process that is driven by clonal selection [92]. This fits the Darwinian process where natural selection occurs based on the survival of the fittest cell population [93]. Many studies have indicated the role of p53 mutations to explain clonal expansion in cancer [78, 79]. In prostate cancer, the same p53 mutations were seen at a lower frequency in the primary tumor and at a higher frequency during metastasis, indicating a clonal expansion of cells harboring specific p53 mutations [94]. Similar observations were seen in brain tumor progression where the same p53 mutations were predominant in low-grade as well as high-grade tumors [95]. In the lower grade tissues, 60% of the cells retained one wildtype p53 allele along with the mutant form, however, in the higher grade tumors, virtually in all the cells, the wildtype p53 allele was lost and only the mutant form was present [95]. These observations suggest that the progression of brain tumors was associated with a clonal expansion of cells acquiring specific missense mutations in the p53 gene, thereby providing a selective growth advantage to the cells [95]. Although several of the p53 mutations are reported to form amyloid since they destabilize faster and carry out the associated function towards tumorigenesis, direct evidence is still lacking to explicitly study the role of p53 amyloid in clonal evolution. Several metastasis models have been suggested which are consistent with the clonal selection hypothesis [96]. One of the models is parallel evolution, which suggests that metastasis can materialize in the early stages of disease progression followed by a parallel evolution of the primary and metastatic tumors (Fig. 4). The parallel evolution hypothesis was coined after observing different phenotypes of gene mutations of breast cancer cells within the bone marrow and that of tumor cells within the primary tumor [96]. In accordance with the above hypothesis, studies were performed with a mouse model expressing amyloid forming p53R245W mutants [97]. When primary tumors and metastases were sequenced, a parallel evolutionary pattern of metastases was observed [97]. Another hypothesis that supports the role of p53 mutation is the clonal dominance model (Fig. 4). It was suggested that when a metastatic subclone occurs within a primary tumor, the cells from this subclone have the ability to surpass and dominate the primary tumor and establish phenotypic similarities between the primary tumor and the metastatic foci [96]. The morphology or phenotype of the p53 amyloids varies depending on the genetic alteration born by the gene [98]. Recent reports have also suggested that the same sequence can result in different amyloid structures, as observed in p53 core domain aggregates [99]. Studies similar to such hypotheses have been conducted to also understand the molecular basis responsible for prion strain diversity, which is of practical relevance in prion diseases [100]. The diversity in the phenotypes between these prion strains is mainly due to the differences in the PrPsc molecule conformation. In Sporadic Creutzfeldt-Jakob disease, methionine at residue 129 is associated with a higher synaptic pattern of PrPsc deposition than the presence of valine at the same position [101, 102] . A recent review has highlighted the diversity in the conformation of the tau protein, existing across the brains of AD patients, leading to diverse clinical phenotypes [103]. It was reported that the reason behind heterogeneity in tau conformation is mainly because of differences in various post-translational modifications like ubiquitination or kinase activity resulting in diverse phosphorylation patterns [103]. The same have also been highlighted in the case of α-Syn fibrils, that they can assemble into polymorphs with different structural and functional/biological activities, thus accounting for diverse disease phenotypes amongst the synucleinopathies [104, 105] All the above studies suggest that these similar phenotypic variations as observed in other prions and prion-like proteins might occur with p53 amyloid/fibrils that might result in clonal expansion (Fig. 4).

## Amyloid/Mutant p53 reactivation towards therapeutics

**Fig. 5:**
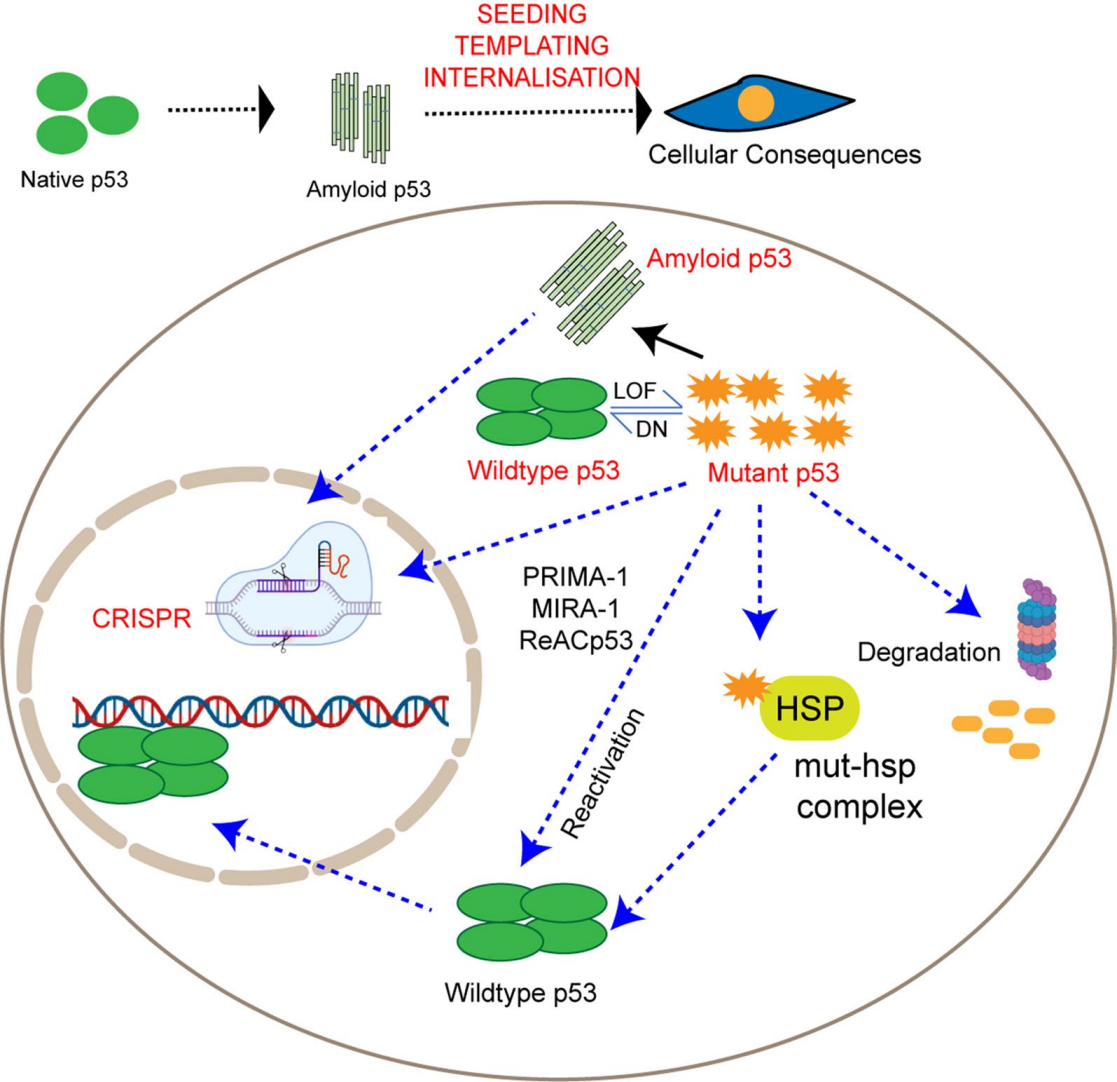
Schematic showing different approaches for reactivation of mutant p53. Different therapeutic approaches are shown in the schematic. By using CRISPR/Cas9 mediated genome editing tools the mutant p53 can be converted to wildtype form. The mutant or amyloid p53 could be reactivated or degraded by small molecule compounds, anti-aggregating compounds, or peptides

Based on all the above instances, it is well evident that mutant p53 and amyloid p53 play a vital role in tumor initiation, progression, and metastasis. Several of the p53 mutants are known to form p53 amyloid and display the LOF and GOF phenotype [10, 19, 23, 91, 106, 107]. The entire road of cancer progression is controlled by p53 via several interacting signaling molecules and pathways. Therefore, any therapeutic approach leading towards p53 reactivation can be of severe importance to the current precision medicine field [5]. Our review further highlights several studies that have hinted at p53 reactivation as a vital step toward therapeutics. Many studies have shown that restoring p53 expression by either direct introduction of recombinant proteins or by chemical/intracellular mediated p53 reactivation can have therapeutic implications (Fig. 5). Synthetic biology tools such as CRISPR have proven beneficial in reactivating p53 and rerouting the cell toward apoptosis. Several compounds have been tested to restore the wild-type p53 conformation and activity in cancer cells, by inhibiting proliferation [108].

### Reactivation of p53 function by small molecules

Previous reports in breast cancer and ovarian cell lines have suggested that PRIMA-1 can reverse mutant p53 aggregate accumulation in cancer cells by substantially decreasing p53 aggregates [109]. Mutant p53-expressing Saos-2 cells also show selective growth-inhibitory and apoptosis-inducing effects when treated with PRIMA-1 (2,2-bis(hydroxymethyl)-1-azabicyclooctan-3-one) [110]. Further, PRIMA-1 and its structural analog PRIMA-1MET (APR-246) can also inhibit human xenograft tumor growth in SCID mice [110]. PRIMA-1 can therefore restore the sequence-specific DNA binding and can activate the functional conformation of mutant p53 in vitro as well as in living cells. PRIMA-1 was observed to rescue both contact and structural p53 mutants [110]. Like PRIMA-1, a maleimide-derived molecule MIRA-1 can also reactivate the DNA binding property of mutant p53 in vitro as well as in cells (Table 1). In SCID mice, a structural homolog of MIRA-3 had anticancer efficacy against human tumour xenografts expressing mutant p53 [111].

**Table 1:** Some major approaches towards p53 reactivation and tumor regression

Synthesized drugs/small molecules			
Drug	Status of p53	Study conclusion	Refs
PRIMA-1	amyloid aggregation of mutant p53	Restore unfolded p53 mutants to a native that can induces apoptosis and activates several p53 target genes	[109]
MIRA-1	Mutant p53	Inhibits viability, colony formation, and migration. Apoptosis of Multiple Myeloma cells irrespective of p53 status sue to upregulation of Puma and Bax along with downregulation of Mcl-1 and c-Myc	[152]
RETRA	Mutant p53	Activates several p53-regulated genes and specifically suppresses mutant p53-bearing tumor cells in vitro and in mouse xenografts	
MB710, aminobenzothiazole derivative	oncogenic p53 mutation Y220C	Binds tightly to the pocket of Y220C mutant and stabilizes p53-Y220C in vitro	[153]
MB725, an ethylamide analogue of MB710	oncogenic p53 mutation Y220C	Increased expression of p53 target genes such as BTG2, p21, PUMA, FAS, TNF, and TNFRSF10B, promoting apoptosis and cell cycle arrest	[153]
PK11007 (2-Sulfonylpyrimidines)	Mutant p53	Unstable p53 reactivation in some cancer cell lines, resulting in upregulation of p53 target genes, p21 and PUMA	[154]
Pramlintide	Null p53	Rapid tumour regression in p53-deficient thymic lymphomas	[155]
PhiKan083, 1-(9-ethyl-9H-carbazole-3-yl)-N-methylmethanamine	Mutant p53	Can binds to a mutant form increasing the melting temperature of the protein to slow down its rate of aggregation	[156]
17-AAG- Inhibitor of HSP90	Mutant p53	Stimulate the heat shock transcription factor (HSF-1) and upregulate the endogenous HSP70 that results in the formation of soluble folding intermediates of cytoplasmic p53 R175H	[157]
Natural products			
Propolis, resinous substance produced by honeybees	Wildtype/mutant	Activation of p53-GADD45 signaling resulting in growth arrest of cancer cells	[158]
Curcumin-based Zn(II)-complex	Mutant p53	Restoring transactivation functions and inducing apoptotic cell death	[159]
Genetic modulation			
Deletion of ΔNp63 and ΔNp73	Null p53	Upregulation of key metabolic regulators such as IAPP, GLS2, and TIGAR thereby increasing apoptosis and tumor regression	[160]

The list of small molecules targeting mutant p53 also includes WR1065, which is a derivative of the cytoprotective drug Amifostine. Amifostine is known to protect normal cells from the toxic effects of irradiation through free-radical scavenging [112]. WR1065 is shown to rescue wild-type p53 via the JNK pathway and DNA damage-independent p53 phosphorylation and stabilization [113]. WR1065 is also observed to partially restore the DNA binding activity of mutant p53 and induce expression of p21, GADD45 resulting in cell cycle arrest [114]. Restoring p53 activity can also be targeted by degrading mutant p53, as observed when cells were treated with capsaicin, one of the major constituents of peppers. Degradation of mutant p53 restored the wild-type p53 functions leading to cell death [115].

Several small molecules have been reported to restore the biological function of p53 (Table 1). Efforts have also been directed to generate some hybrid anticancer drugs that can rescue two or more targets simultaneously. One such class of drugs are piperlongumine (PL) derivatives with an aryl group inserted at the C-7 position. These compounds displayed antiproliferative properties against different cancer cell lines but displayed more cytotoxicity against the SKBR-3 breast cancer cells, which harbour a hotspot p53 mutation, R175H [116].

A styrylquinazole, CP-31398, was shown to restore the DNA-binding ability of mutant p53 [108, 117. Treatment with this drug resulted in tumor growth reduction in urothelial cancer [118]. In a separate study CP-31398 induced cell growth inhibition, apoptosis, and autophagy in pancreatic adenocarcinoma cell lines by activating p53 phosphorylation (S15) and the DNA binding ability of p53 [119].

A derivative of CP-31398, STIMA-1, is observed to display growth defect in H1299 and Saos-2 cells expressing mutant p53 R175H and R273H respectively [120]. STIMA-1 treatment also induced the DNA-binding ability of mutant p53 thereby inducing the expression of various downstream targets of p53 [120].

Several p53 mutants were shown to restore their functions in the presence of a phyto-alkaloid, Ellipticine (5,11-dimethyl-6H-pyrido[4,3-b]carbazole) [117]. Ellipticine also resulted in the upregulation of p21 gene [121]. Ellipticine has been reported to prevent the growth of the HCC cell line, HepG2 which expresses a WT p53 [121]. Additionally, ellipticine treatment also enhanced mitochondrial p53 [122] and initiated the mitochondrial apoptotic pathway [121].

Another compound, PK7088, was shown to bind the Y220C p53 mutant in cancer cells, resulting in cell-cycle arrest, growth inhibition, and apoptosis [123]. Nutlin-3, an MDM2 inhibitor, and PK7088 collaborate to increase the quantity of folded p53. By interacting with p53 pockets or alkylating thiols, chemicals based on the Nutlin structure are intended to reactivate mutant p53 [124]. Nutlin-3 can arrest proliferating cancer cells and induce apoptosis in numerous cell lines like breast, melanoma, lung, colorectal, and renal cancer, which have mostly wild-type p53 [125]. Another synthetic small molecule that can target MDM2-p53 interaction is MI-319, which binds to MDM2 and can suppress cell cycle growth and induce apoptosis as observed in pancreatic cancer [126]. Thus, the re-activation of p53 by specific MDM2 inhibitors could be another promising therapeutic strategy towards cancer suppression. Other examples include a number of chemical classes like imidazoles, pyrrolidinones, spiro-oxindoles, benzodiazepinedione derivatives, and substituted piperidine derivatives (e.g., Nutlins or RG7112). Compounds such as spiro[3Hindole-3,2′-pyrrolidin]-2(1H)-one derivatives can also inhibit MDM2-p53 interactions thereby stabilizing p53 [127].

A number of maleimide compounds can restore mutant p53's ability to bind DNA while maintaining its active conformation [107]. It has been demonstrated that PK5174, a related substance, can stop Y220C from aggregating. To restart p53 function, several substances, including RITA, demonstrate a variety of modes of action [128]. It can target various p53 mutants, R273H, R175H, R280K and R248W by inhibiting MDM2-p53 interactions and restoring the transcriptional activity of the mutant protein [107].

A series of small molecules, α-helical mimetics, and oligopyridylamides, were reported to inhibit the formation of amyloid associated with type II diabetes and Alzheimer’s disease [129]. Recently, a similar observation was made in peptides corresponding to p53 residues (248–273) of WT p53 DBD, and another composed of the same sequence but harboring the R248W mutation [129]. The effects of 10 compounds (ADH-1–10) on the aggregation of peptide harbouring R248W mutation were studied. Out of the 10 compounds screened, ADH-6 completely inhibited the peptide’s amyloid formation and could convert the insoluble cytosolic mutant p53 aggregates into soluble protein [129]. Therefore, small molecule inhibitors can serve as potent anticancer agents in the case of amyloid p53-mediated cancers.

During DNA damage, p53 is activated as a result of several posttranslational modifications occurring mostly at the amino and carboxyl-terminal regions of the protein [125, 126]. Another posttranslational modification is acetylation, which involves covalent modification of p53, which is observed in response to DNA damage [130]. Previous studies have shown that the histone acetyltransferase, p300/CBP favours p53-dependent transcriptional activation [131, 132]. Moreover, acetylation of p53 by p300 also stimulates DNA-binding activity and transcriptional functions [127, 133, 134, 135]. Acetylated p53 is shown to increase when the cells are treated with Histone deacetylases (HDAC) inhibitors, thus preventing p53 degradation under in vivo conditions [131].

### Reactivation of p53 function by peptides

Peptides have been screened to identify candidates which can favor the correct conformation of p53, thereby restoring p53 functions [136, 137]. These peptides, when transfected in cell lines, display enhanced cell death and DNA binding ability of p53 [138]. Tumor xenograft assays showed complete suppression of the tumor in some cases, and reduction in tumor size in others [138].

In previous reports, ReACp53 is reported to be a cell-penetrating peptide that is intended to rescue p53 activity in cancer cell lines and organoids produced from high-grade serous ovarian carcinomas and by suppressing the production of p53 amyloid (HGSOC) [139].

Another synthetic peptide (peptide 46), corresponding to the C-terminal residues of p53 (361–382), has shown reactivation of and restoration of p53 transcriptional activity in some mutant p53 such as in the case of R273H [140]. The peptide when transfected in Saos-2 cells, expressing a p53 mutant R273H, displayed cell-cycle inhibition and apoptosis. Furthermore, similar effects on other human cancer cell lines expressing mutant or wild-type p53 were also observed in the presence of peptide 46 [140]. A nine-residue peptide, CDB3, was shown to bind the core domain of p53 leading to its stabilization under in vitro conditions [136–141]  . CDB3 can restore several p53 mutants into wild-type conformation and thus retain their DNA binding ability [117–116].

### Reactivation of p53 function by microRNAs

The miRNAs, discovered in 1993, are generally 19–25 bases in length and do not code for proteins. miRNAs are either overexpressed and act as oncogenes in cancer tissues or they are under-expressed and can suppress proliferation by functioning as tumor suppressors [142]. miRNA regulation can affect the expression of both wildtype and mutant p53 since those targeting p53 are not able to distinguish between wildtype or mutant form if the target site is not the mutation site [142]. Unless a miRNA specifically targets a mutated region of p53 mRNA it is unable to distinguish between the wild-type and mutant versions of the mRNA. Some of the miRNAs can regulate the stability of the p53 protein by targeting negative regulators, E3 ubiquitin ligase, Mdm2 [143] or an Mdm2-related protein—Mdm4 [144]. This results in p53’s escape from degradation and its stability in the cell [145]. Reports have shown that miR-192/194/215, miR-143/145, miR-29b, miR-32, miR-605, miR-25, miR-32, miR18b, and miR-339-5p were reported to repress MDM2 and activate p53. However, whether miRNAs can directly bind to amyloid or mutant p53 and activate its function is not known.

### CRISPR Cas9 mediated activation

The p53 pathway can be thus activated in response to CRISPR/Cas9 editing, which relies on double stranded breaks [146]. Cas9 is frequently inserted into cell lines when using the CRISPR/Cas9 method for genome editing (Fig. 5). Both TP53-WT and TP53-mutant cell lines that expressed Cas9 showed upregulation of the p53 pathway when Cas9 was introduced. DNA repair was increased as a result of p53 pathway upregulation [147]. The CRISPR/Cas9 method has been demonstrated to rectify a mutant p53 (Tp53414delC) in a human prostate cancer cell line [148]. By combining a Cas9 nickase with cytidine deaminase and uracil DNA glycosylase inhibitor (UGI) proteins to help the mutant CG pair to TA base pair conversion, the TP53 Tyr163Cys mutation in HCC1954 breast cancer cells has been rectified. Base editors, which may instantly change one base or base pair into another to achieve the desired base without producing as many extra undesirable editing byproducts as CRISPR-based techniques do, are another efficient gene editing approach [149]. These methods may be used in the future in p53 reactivation therapy.

## Conclusion and future prospective

The biological activities of p53 are inactivated in almost all malignancies [12, 150]. Several supporting pieces of evidence have proven that p53 aggregation and amyloid formation are among the causes of the protein's loss of function [12, 29, 106, 151]. p53 gain-of-function is known to be associated with tumorigenesis, cancer progression, and metastasis of tumor cells. Numerous studies have supported the fact that the disruption of epithelial cell integrity that contributes to the spreading of cells from solid tumors to adjacent areas, is due to p53 loss and gain of functions by p53 mutant or p53 amyloids. The emerging data from several groups have also highlighted the role of mutant p53 in the acquisition of cancer stemness which results in cancer relapse. We emphasize the need for detailed study of amyloid p53, cell-to-cell transmission mechanism, and identification of new biomarkers or altered pathways. It is necessary to investigate the precise mechanism of p53 prion transfer. Several of the GOF events are well-studied for mutant p53. Now, this is quite important to explore the role of amyloid p53 in GOF leading to EMT, cancer stemness, drug resistance and relapse in human malignancies. For instance, we did not come across any study that investigated the association of the p53 amyloid with non-coding RNAs. This is a novel area that needs to be explored to understand important regulations and might be important to develop novel therapeutic against amyloid p53 in human malignancies. Further, a significant active area of therapeutic intervention in cancer is restoring p53 activity. p53 functional restoration has been carried out in quite a few studies. Currently, emerging research suggests that loss of p53 function because of amyloid accumulation contribute to cancer progression, stemness and resistance in few cancers. This need to be systematically investigated in the patients where p53 is non-function due to amyloid formation and independent of *TP53* mutations. The use of small molecules to degrade aggregate p53 and/or chaperones to refold the misfolded p53 should be developed. The review highlights all the areas where studies are needed to understand the effect of p53 amyloid on cancer progression. Distinguishing between tumors with loss of p53, mutated gain-of-function phenotype, and tumors with amyloid fibrils may help to predict tumor behaviour and help clinicians in their treatment decisions.

## Data Availability

Not applicable.
